# Lactoferrin is required for early B cell development in C57BL/6 mice

**DOI:** 10.1186/s13045-021-01074-6

**Published:** 2021-04-07

**Authors:** Lingyu Wei, Can Liu, Jia Wang, Xiang Zheng, Qiu Peng, Qiurong Ye, Zailong Qin, Zhengshuo Li, Xiaoyue Zhang, Yangge Wu, Yuqing Wen, Xuemei Zhang, Qun Yan, Jian Ma

**Affiliations:** 1 Hunan Key Laboratory of Cancer Metabolism, Hunan Cancer Hospital and the Affiliated Cancer Hospital of Xiangya School of Medicine, Central South University, Changsha, China; 2 Key Laboratory of Carcinogenesis and Cancer Invasion of the Chinese Ministry of Education, NHC Key Laboratory of Carcinogenesis, Cancer Research Institute, School of Basic Medical Science, Central South University, Changsha, China; 3 Department of Pathology, Heping Hospital Affiliated to Changzhi Medical College, Department of Immunology, Changzhi, Shanxi China; 4 Hunan Key Laboratory of Nonresolving Inflammation and Cancer, Hunan Key Laboratory of Translational Radiation Oncology, Changsha, China; 5 Department of Pathology, Affiliated Hospital of Guilin Medical University, Guilin, Guangxi China; 6 Department of Pathology, People’s Hospital of Guanxi Zhuang Autonomous Region, Nanning, Guangxi China; 7 Genetic and Metabolic Central Laboratory, Maternal and Child Health Hospital of Guangxi Zhuang Autonomous Region, Nanning, Guangxi China; 8 Department of Clinical Laboratory, Xiangya Hospital, Central South University, Changsha, China

**Keywords:** Lactoferrin, B cell development, CXCL12, Pre-pro-B cell, Pro-B cell, Systemic lupus erythematosus

## Abstract

Lactoferrin (Lf) is widely distributed in mammalian milk, various tissues, and their exocrine fluids and has many physiological functions, such as bacteriostasis, antivirus, and immunoregulation. Here, we provide evidence that lactoferrin is required for early stages of B cell development in mice. Lactoferrin-deficient (*Lf*^−/−^) C57BL/6 mice showed systematic reduction in total B cells, which was attributed to the arrest of early B cell development from pre-pro-B to pro-B stage. Although the *Lf*^−/−^ B cell “seeds” generated greater pro-B cells comparing to wild type (WT) littermates, the *Lf*^−/−^ mice bone marrow had less stromal cells, and lower CXCL12 expression, produced a less favorable “microenvironment” for early B cell development. The underlying mechanism was mediated through ERK and AKT signalings and an abnormality in the transcription factors related to early differentiation of B cells. The *Lf*^−/−^ mice also displayed abnormal antibody production in T cell-dependent and T cell-independent immunization experiments. In a pristane-induced lupus model, *Lf*^−/−^ mice had more serious symptoms than WT mice, whereas lactoferrin treatment alleviated these symptoms. This study demonstrates a novel role of lactoferrin in early B cell development, suggesting a potential benefit for using lactoferrin in B cell-related diseases.

**Supplementary Information:**

The online version contains supplementary material available at 10.1186/s13045-021-01074-6.

**To the editor,**

Lactoferrin (LF) is widely existed in mammalian milk, neutrophils particles and various tissues and their exudates. It plays a protective role of the body through a variety of functions, such as anti-infection, anti-oxidation, and immune modulation [1, 2]. By constructing a *lactoferrin* gene knockout (*Lf*^*−/−*^) C57BL/6 mice [3, 4], here we investigated the effect of lactoferrin deficiency on B cell development.

Proportion of CD45^+^, T, and dendritic cells remained fairly consistent, but proportion of total B cells of *Lf*^*−/−*^ mice were significantly lower than that of WT controls (Fig. 1a, Additional file 2 Fig. S1). Lactoferrin deficiency did not cause an increase of B cell apoptosis (Fig. 1b). Proportion of hematopoietic progenitor cells displayed little difference between WT and *Lf*^*−/−*^ (Fig. 1c, d). Proportion of pro-B, pre-B and immature B cells in bone marrow of *Lf*^*−/−*^ mice were all significantly lower, whereas proportion of pre-pro-B cells was higher than that of WT (Fig. 1e, f), implying that lactoferrin deficiency inhibited the transition from pre-pro-B to pro-B stage. mRNA expression levels of *lactoferrin* are dynamic in developing B cells, peaking at the pre-pro-B stage (Fig. 1g).

**Fig. 1:**
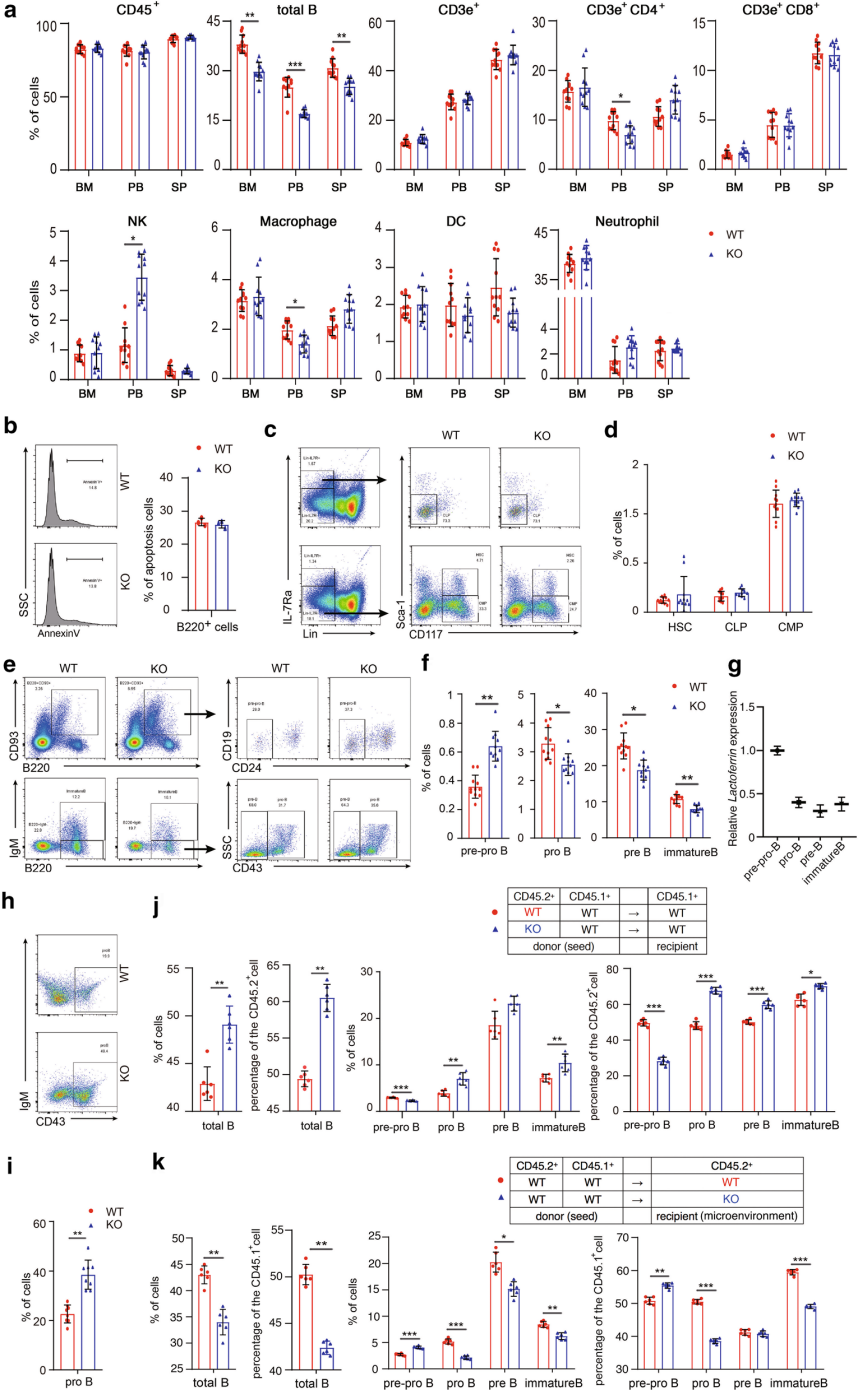
The defect of B cell development in *Lf*^*−/−*^ mice is both cell autonomous and is associated with the bone marrow microenvironment. **a** Lactoferrin deficiency leads to imbalance of hematopoiesis. Cells were isolated from the bone marrow (BM), peripheral blood (PB), and spleens (SP) of *Lf*^*−/−*^ mice and WT littermates. Frequencies of indicated cells were identified by flow cytometry. All immune cells were firstly gated on CD45^+^. Each group has 11 mice. **b** Splenic B cells were sorted from WT and *Lf*^*−/−*^ mice. The amount of apoptosis cells was detected by flow cytometer. Representative data from three independent experiments are shown. **c**, **e** Representative strategy of flow analysis of **c** HSC, CLP, CMP and **e** pre-pro-B, pro-B, pre-B, immature B cells. Cells were isolated from the mice bone marrow. **d**, **f** Frequencies of **d** HSC (Lin^−^ IL7R^−^ C-kit^+^ Sca-1^+^), CLP (Lin^−^ IL7R^+^ C-kit^lo^ Sca-1^lo^), CMP (Lin^−^ IL7R^−^ C-kit^+^ Sca-1^−^) and **f** pre-pro-B (AA4.1^+^B220^+^CD19^−^CD24^−^), pro-B (B220^+^CD43^+^IgM^−^), pre-B (B220^+^CD43^−^IgM^−^), immature B (B220^+^IgM^+^) cells were identified by flow cytometry. Each group has 11 mice. (note, CD117 is C-kit, CD93 is AA4.1.) **g** mRNA expression of *lactoferrin* in distinct stages of developing B cells from WT mouse bone marrow was evaluated by RT-qPCR. **h**, **i** In vitro B cell differentiation experiment: purified pre-pro-B cells from *Lf*^*−/−*^ or WT mice were cocultured with OP9 stromal cells in the presence of IL-7 (10 ng/ml), SCF (5 ng/ml), and Flt3L (5 ng/ml) for 9 days. **h** Representative data from eight specimens each group are shown. **i** The proportions of pro-B cells generated were then determined by flow cytometric analysis. **j** Bone marrow transplantation experiment: bone marrow cells from either WT or *Lf*^*−/−*^ (CD45.2^+^) mice with bone marrow from syngenic mice (CD45.1^+^) were mixed at a 1:1 ratio. The recipient WT mice (CD45.1^+^) were irradiated in fractionated doses (5 Gy × 2), and 16 h later, the recipient mice were injected with mixed cells (2 × 10^6^ cells). After 6 weeks, the recipient mice were killed to prepare the bone marrow single-cell suspension, and the B cell proportion of each stage of B cell differentiation was analyzed by flow cytometry. Representative data from six mice each group are shown. **k** Bone marrow transplantation experiment: bone marrow cells from CD45.2^+^ WT mice or from CD45.1^+^ WT mice were mixed at a 1:1 ratio, while WT or *Lf*^*−/−*^ mice with CD45.2^+^ genetic background were used as recipient mice. The rest of the operation was the same as j. Representative data from six mice each group are shown

Contrary to our expectations, the capability of pre-pro-B to generate pro-B cells was significantly higher in *Lf*^*−/−*^ group than WT both in vitro (Fig. 1h, i) and in vivo (Fig. 1j, Additional file 3 Fig. S2), implying that *Lf*^*−/−*^ “seeds” generated more pro-B cells comparing to WT “seeds”. We analyzed the global transcriptome change of pre-pro-B and pro-B cells from WT and *Lf*^*−/−*^ mice. GO, KEGG, and GSEA [5] reveal the signaling differences between them (Additional file 4 Fig. S3, Additional file 5 Fig. S4). Transcription factors Pu.1, Bcl11a, E2A, Ebf1, and Pax5 regulate the differentiation of common lymphoid progenitor (CLP) cells into B cell lineage [6]. Expression levels of *Pax5*, *Ebf1* and *Tcf3* in *Lf*^*−/−*^ pre-pro-B and pro-B cells were higher than WT (Additional file 4 Fig. S3F, Additional file 5 Fig. S4E).

In another side, in vivo bone marrow transplantation experiment revealed that lactoferrin deficiency in bone marrow microenvironment retards the transition from pre-pro-B to pro-B stage (Fig. 1k). So, the dysregulation of early B cell development in lactoferrin-deficient mice is both cell autonomous and is associated with bone marrow microenvironment.

Proportion of stromal cells in *Lf*^*−/−*^ bone marrow were decreased significantly comparing to WT (Fig. 2a). CXCL12 expressions were significantly lower in *Lf*^*−/−*^ bone marrow stromal cells but not the IL-7 expressions (Fig. 2b–d). Lactoferrin can modulate *Cxcl12* expression in mouse stromal cell OP9 (Additional file 6 Fig. S5A, B); and CXCL12 protein promoted early B cell differentiation (Fig. 2e). CXCL12 modulate the differentiation of lymphocytes by binding to its receptor CXCR4 [7]. Expression of CXCR4 in B cells isolated from *Lf*^*−/−*^ mice bone marrow was higher than WT (Fig. 2f, Additional file 6 Fig. S5C). CXCR4 inhibitor suppressed B cells differentiation, while CXCL12 can promote it (Fig. 2g). The CXCL12-CXCR4 axis transmits signals through MAPK and AKT signalings [8]. CXCR4 inhibitor reduced the phosphorylation levels of AKT and ERK, whereas CXCL12 increased them (Fig. 2h, Additional file 6 Fig. S5D). AKT or ERK inhibitors suppressed B cell differentiation, while CXCL12 can reverse their effects (Fig. 2i). So, *Lf*^*−/−*^ mouse bone marrow stromal cells were not conducive to the early B cell development compared with WT controls (Fig. 2j).

**Fig. 2:**
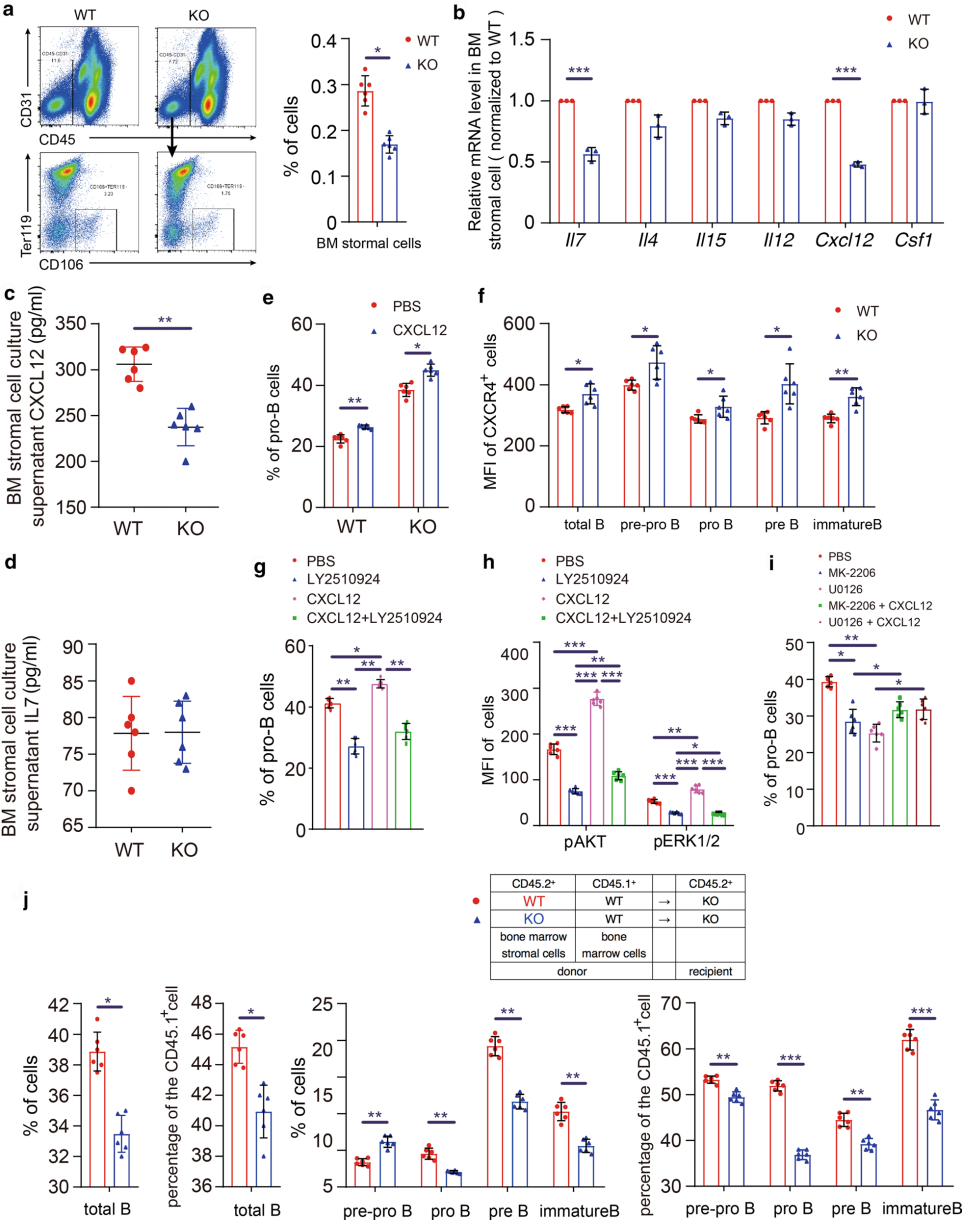
Lactoferrin deficiency reduces the proportion of stromal cells in the bone marrow microenvironment and decreases CXCL12 expression to inhibit the early development of B cells. **a** Cells were isolated from the bone marrow of *Lf*^*−/−*^ mice and WT littermates. Frequencies of bone marrow stromal cells (CD45^−^CD31^−^TER119^−^CD106^+^) were identified by flow cytometry. Representative data from 11 specimens each group are shown. **b** Expression levels of some factors in bone marrow stromal cells sorting from WT and *Lf*^*−/−*^ mice were determined by RT-qPCR. n = 3. **c**, **d** ELISA was performed for CXCL12 and IL7 of the supernatant of bone marrow stromal cells (isolated from WT and *Lf*^*−/−*^ mice). **e** In vitro differentiation experiment: bone marrow cells from *Lf*^*−/−*^ or WT mice were added in 12-well plates, with CXCL12 recombinant protein (10 ng/ml), or PBS as control, for 9 days. The proportions of pro-B cells generated from the bone marrow cells were then determined by flow cytometric analysis. **f** Different stages of B cells were isolated from the WT and *Lf*^*−/−*^ mouse bone marrow cells, and the surface expression of CXCR4 in each stage of B cells was determined by flow cytometric analysis. Each group has six mice. **g, h** In vitro differentiation experiment: bone marrow cells from *Lf*^*−/−*^ mice were added in 12-well plates at 5 × 10^4^ cells per well, with (1) PBS, or (2) LY2510924 (10 ng/ml), or (3) CXCL12 recombinant protein (10 ng/ml), or (4) LY2510924 (10 ng/ml) and CXCL12 recombinant protein (10 ng/ml), for 9 days. Each group has six specimens. The proportions of pro-B cells (**g**) and the phosphorylation levels of AKT and ERK in total B cells (**h**) generated from the bone marrow cells were then determined by flow cytometric analysis. **i** In vitro differentiation experiment: bone marrow cells from *Lf*^*−/−*^ mice were added in 12-well plates at 5 × 10^4^ cells per well, with (1) PBS, or (2) MK-2206 2HCL (10 ng/ml), or (3) U0126-EtOH (10 ng/ml), or (4) MK-2206 2HCL (10 ng/ml) and CXCL12 (10 ng/ml), or (5) U0126-EtOH (10 ng/ml) and CXCL12 (10 ng/ml), for 9 days. The proportions of pro-B cells generated from the bone marrow cells were then determined by flow cytometric analysis. **j** Bone marrow stromal cells transplantation experiment: bone marrow stromal cells from WT or *Lf*^*−/−*^mice (CD45.2^+^) were mixed with bone marrow cells from WT (CD45.1^+^) mice at a ratio of 1:3, and then the mixed cells were transplanted into the irradiated lethal *Lf*^−/−^ mice (CD45.2^+^). After 6 weeks, the recipient mice were killed to prepare the bone marrow single-cell suspension, and the B cell proportion of each stage of B cell differentiation was analyzed by flow cytometry

Dysregulation of difference type B cells were observed in the spleen of *Lf*^*−/−*^ mice comparing to WT (Additional file 7 Fig. S6A, B). Under the stimulation of TD and TI antigens, lactoferrin deficiency led to a decrease in proportion of total, follicular and T1 B cells (Additional file 7 Fig. S6C). The TNP-specific IgM and IgG2a, 2b responses were higher in *Lf*^*−/−*^ mice (Additional file 7 Fig. S6D, E).

Systemic lupus erythematosus (SLE) is associated with a number of immunomodulatory abnormalities including B cell dysfunction [9]. In a pristane-induced SLE mouse model, the degree of injury to the glomerular filtration barrier, kidney damage, glomerular deposits of IgG antibodies, serum levels of IgM and IgG2b, amounts of anti-dsDNA total IgM and IgG in WT mice was lower than that in *Lf*^*−/−*^ mice, and oral lactoferrin treatment alleviated the symptoms (Additional file 8 Fig S7). Lactoferrin plays a protective role in the SLE model.

In conclusion, this study demonstrates that lactoferrin is required for the early development of B cells in C57BL/6 mice by regulating the microenvironment of bone marrow stroma through CXCL12 release. *Lf*^*−/−*^ mice had more severe symptoms in a SLE model, which can be alleviated by oral administration of lactoferrin, and that was related to the dysfunction of B cells induced by lactoferrin deficiency. Lactoferrin may be applied in preventative medicine or nutrition supplies for B cell-related diseases.

## Supplementary Information

**Additional file 1:** Methods.

**Additional file 2: Fig. S1.** Representative flow analysis diagrams for analysis of the different hematopoietic cells.

**Additional file 3: Fig. S2.** Representative flow analysis diagrams of in vivo bone marrow transplantation experiment.

**Additional file 4: Fig. S3.** Lactoferrin deficiency alters genes expression profile and key pathways in pre-pro-B cells.

**Additional file 5: Fig. S4.** Lactoferrin deficiency alters genes expression profile and key pathways in pro-B cells.

**Additional file 6: Fig. S5.** Effect of lactoferrin deficiency on Cxcl2 expression.

**Additional file 7: Fig. S6.** Lactoferrin deficiency affects the proportion of splenic B cells subclasses and antibody production in B cells.

**Additional file 8: Fig. S7.** Lactoferrin deficiency promotes the progression of SLE in mice.

**Additional file 9: Table S1.** Information on the antibodies used in FACS or mice experiments.

**Additional file 10: Table S2.** Antibodies information for ELISA.

**Additional file 11: Table S3.** Primer sequences used for qPCR.

## Data Availability

All data generated or analyzed during this study are included in this published article. The RNA-seq raw expression files and details have been deposited in NCBI GEO under accession number GSE163097.

## Abbreviations

CLP: Common lymphoid progenitor
CMP: Common myeloid progenitor
ELISA: Enzyme-linked immunosorbent assay
HSC: Hematopoietic stem cell
LF: Lactoferrin
SLE: Systemic lupus erythematosus
TI: T cell independent
TD: T cell dependent
WT: Wild type
