# Supplementary material for: Lactoferrin is required for early B cell development in C57BL/6 mice
Source: J Hematol Oncol. 2021 Apr 7;14:58. doi: 10.1186/s13045-021-01074-6 (PMC8028198; doi:10.1186/s13045-021-01074-6)
Supplement: Supplementary file 1 — Additional file 1: Methods. [file 13045_2021_1074_MOESM1_ESM.pdf]

# Lactoferrin is required for early B cell development in C57BL/6 mice

## Additional Files

### Additional file 1. Methods

***Mice***

The generation of *lactoferrin* knockout mice ($Lf^{-/-}$) was previously reported by our group [1]. We maintained $Lf^{-/-}$ mice on a C57B/L6 background and all mice were housed in a specific pathogen-free facility, and age matched littermates were used as controls in this study. This study was carried out in strict accordance with the recommendations in the Guide for the Care and Use of Laboratory Animals (National Institutes of Health Publications No. 8023, revised 1978). The protocol was approved by the Animal Ethics Committee of Central South University (China). All surgeries were performed under sodium pentobarbital anesthesia, and all efforts were made to minimize animal suffering.

***Isolation of mononuclear cells***

Isolation of mononuclear cells were previously described [2].

***Flow cytometry analysis and sorting***

Flow cytometry analysis and sorting were previously described [3]. The following fluorescein-conjugated anti-mouse antibodies (Additional file 9 Table S1 for antibodies information) were used.

***In vitro B cell differentiation experiment***

Purified pre-pro-B cells from $Lf^{-/-}$ and WT mice were added in 12-well plates at $5\times10^4$ cells per well and cocultured on OP9 stromal cells in the presence of IL-7 (10 ng/mL), SCF (5ng/mL), and Flt3L (5 ng/mL) (PeproTech, NJ, USA) for 9 days. The frequencies of pro-B cells generated were then determined by flow cytometric analysis.

***In vivo bone marrow transplantation experiment***

*(1) Bone marrow cell transplantation experiment* (for Fig. 1J, K): two genetic backgrounds bone marrow cells were mixed at a 1:1 ratio, one was from CD45.2$^{+}$ mice, the another was from syngenic mice (CD45.1$^{+}$). The recipient mice (either CD45.1$^{+}$ or CD45.2$^{+}$) were irradiated in fractionated doses (5Gy×2), and 16 hours later, the mice were injected with mixed cells (2×10$^{6}$ cells).

*(2) Bone marrow stromal cell transplantation experiment* (for Fig. 2J): CD45.2$^{+}$ WT or *Lf*$^{-/-}$ mice bone marrow stromal cells were mixed with CD45.1$^{+}$ WT mice bone marrow cells at a 3:1 ratio. The recipient *Lf*$^{-/-}$ mice (CD45.2$^{+}$) were irradiated in fractionated doses (5Gy×2), and 16 hours later, the mice were injected with mixed cells (2×10$^{6}$ cells).

After 6 weeks, the recipient mice were sacrificed to prepare the bone marrow single-cell suspension, and the B cell proportion of each stage of B cell differentiation was analyzed by flow cytometry.

***T cell-independent (TI) and T cell-dependent (TD) antibody immunization***

*(1) TI antibody immunization*: mice were intraperitoneal injected (i.p.) with 50 μg TNP-Ficoll (Biosearch Technologies, CA, USA). Mice serum samples at day 0, 3, 7 and 10 post-immunization were analyzed by ELISA for determination of TNP-specific immune-globulins.

*(2) TD antibody immunization*: mice were i.p. with 100 μg TNP-KLH (Biosearch Technologies), and i.p. with same dose of TNP- KLH at days 14 for secondary immunization. Mice serum samples at days 0, 7, 14 and 28 post-immunization were analyzed by ELISA for determination of TNP-specific immune-globulins.

***Pristane-induced SLE mouse model***

Female *Lf*$^{-/-}$ or WT mice were randomly divided into three groups: (1) normal control group mice received 0.5 ml of saline solution (0.9% NaCl); (2) SLE group: mice received a single dose of 0.5 ml of pristane (Sigma-Aldrich, MO, USA); (3)

lactoferrin treatment group mice received 0.5 ml of pristane once and received daily lactoferrin (200 mg/kg bodyweight, dissolved in water, Sigma-Aldrich) for six months. Then mouse urine was collected every other month and the urine protein content was measured using the Albustix test paper (Bayer, Germany). Six months later, all mice were sacrificed and the kidneys were removed for morphometrical, immunofluorescence and biochemical analysis.

***Enzyme-linked immunosorbent assay (ELISA)***

ELISA was previously described [4]. Additional file 10 Table S2 for antibodies information.

***Immunofluorescence***

After fixed and washed with acetone, the mouse kidney frozen sections were incubated with FITC-goat anti-mouse IgG (Kangwei, Beijing, China) in dark for 30 minutes, and the sealed slices were observed under a fluorescence microscope.

***Reverse transcription and quantitative real-time PCR***

RT-qPCR was previously described [2]. All reported results were the average ratios of three independent experiments. The primers used for qPCR are listed in Additional file 11 Table S3.

***RNA sequencing***

The pre-pro-B cells and pro-B cells were isolated from the WT and *Lf*$^{-/-}$ mice (each group has three mice) by flow cytometry. Then the cell RNA was sequenced by the NovaSeq 6000 high-throughput sequencing service (Jingneng, Shanghai, China). Data were extracted and normalized according to the manufacturer's standard protocol. The RNA-seq raw expression files and details have been deposited in NCBI GEO under accession number GSE163097. Log-fold changes of up- or downregulated genes in *Lf*$^{-/-}$ mice were selected with a significance threshold of $p < 0.05$. GO enrichment and KEGG pathway enrichment analysis of differently expressed genes were,

respectively, performed using R based on the hypergeometric distribution. Hierarchical cluster analysis of differently expressed genes was performed to explore genes expression pattern.

### *Statistical analysis*

Statistical analysis was performed using SPSS17.0 and GraphPad Prism 8, with Student's t test used for two-group comparisons and one-way analysis of variance (ANOVA) for multigroup analyses. Data were presented as means ± SEM of replicate experiments. The differences were considered statistically significant at $P < 0.05$ ($^{*}P < 0.05$, $^{**}P < 0.01$, and $^{***}P < 0.001$).

## References


1. Ye Q, Zheng Y, Fan S, Qin Z, Li N, Tang A, Ai F, Zhang X, Bian Y, Dang W, et al: Lactoferrin deficiency promotes colitis-associated colorectal dysplasia in mice. *PLoS One* 2014, 9:e103298.
2. Wei L, Zhang X, Wang J, Ye Q, Zheng X, Peng Q, Zheng Y, Liu P, Zhang X, Li Z, et al: Lactoferrin deficiency induces a pro-metastatic tumor microenvironment through recruiting myeloid-derived suppressor cells in mice. *Oncogene* 2020, 39:122-135.
3. Li Z, Wang J, Zhang X, Liu P, Zhang X, Wang J, Zheng X, Wei L, Peng Q, Liu C, et al: Proinflammatory S100A8 Induces PD-L1 Expression in Macrophages, Mediating Tumor Immune Escape. *J Immunol* 2020, 204:2589-2599.
4. Zhang X, Wei L, Wang J, Qin Z, Wang J, Lu Y, Zheng X, Peng Q, Ye Q, Ai F, et al: Suppression Colitis and Colitis-Associated Colon Cancer by Anti-S100a9 Antibody in Mice. *Front Immunol* 2017, 8:1774.
