# Supplementary material for: Lactoferrin is required for early B cell development in C57BL/6 mice
Source: J Hematol Oncol. 2021 Apr 7;14:58. doi: 10.1186/s13045-021-01074-6 (PMC8028198; doi:10.1186/s13045-021-01074-6)
Supplement: Supplementary file 2 — Additional file 2: Fig. S1. Representative flow analysis diagrams for analysis of the different hematopoietic cells. [file 13045_2021_1074_MOESM2_ESM.pdf]

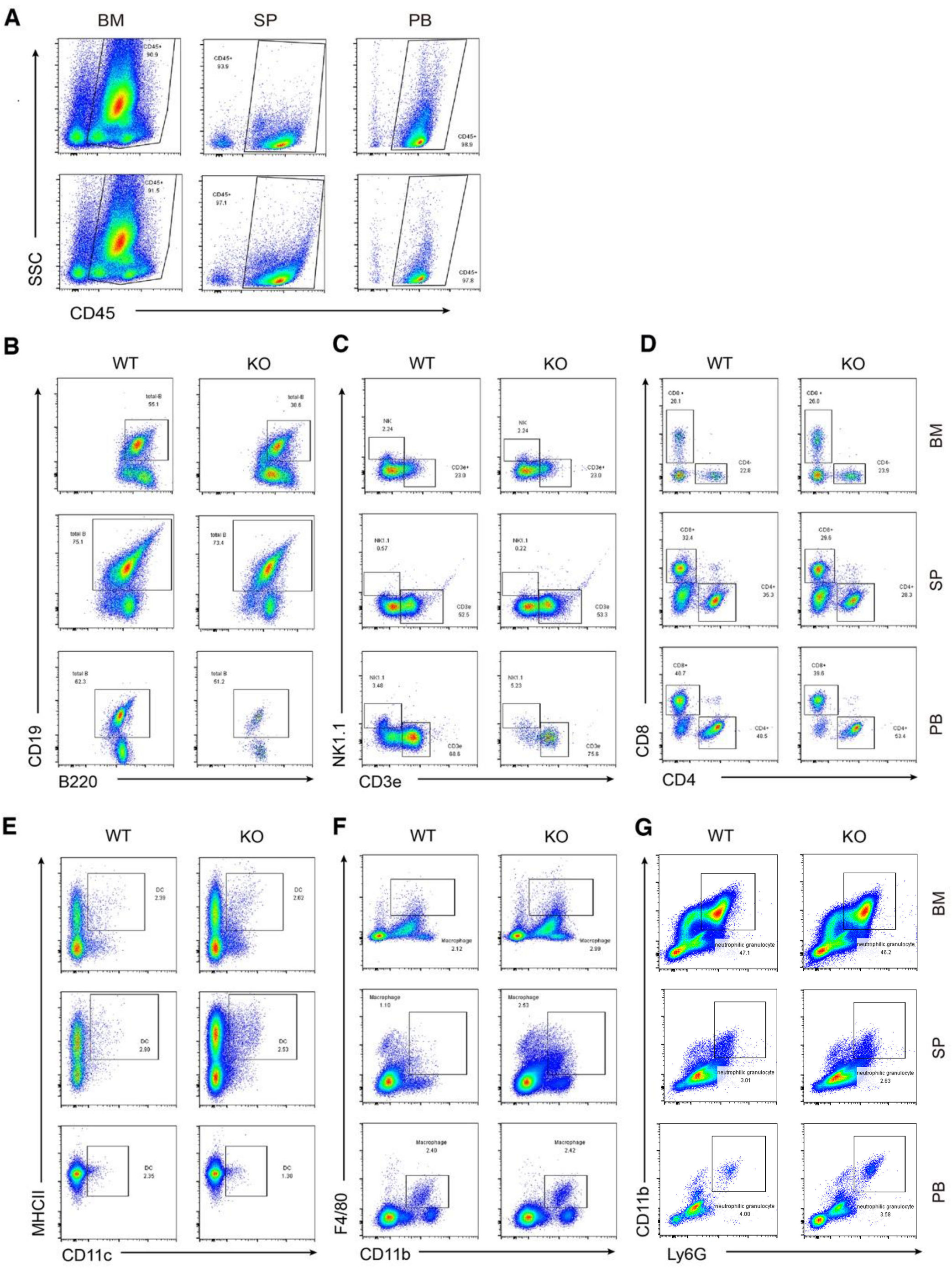

Additional file 2. Fig. S1. Representative flow analysis diagrams for analysis of the different hematopoietic cells (supplementary for Fig. 1). Cells were isolated from the bone marrow (BM), peripheral blood (PB) and spleens (SP) of $Lf^{-/-}$ mice and WT littermates. **(A)** $CD45^{+}$cells, **(B)** total B cells ($CD19^{+}B220^{+}$), **(C)** total T cells ($CD3e^{+}$), NK cells ($NK1.1^{+}$), **(D)** $CD4^{+}$T cells, $CD8^{+}$T cells, **(E)** DC ($MHCII^{+}CD11c^{+}$), **(F)** macrophage ($F4/80^{+}CD11b^{+}$), **(G)** neutrophil granulocytes ($CD11b^{+}Ly6G^{+}$).
