# Supplementary material for: Lactoferrin is required for early B cell development in C57BL/6 mice
Source: J Hematol Oncol. 2021 Apr 7;14:58. doi: 10.1186/s13045-021-01074-6 (PMC8028198; doi:10.1186/s13045-021-01074-6)
Supplement: Supplementary file 3 — Additional file 3: Fig. S2. Representative flow analysis diagrams of in vivo bone marrow transplantation experiment. [file 13045_2021_1074_MOESM3_ESM.pdf]

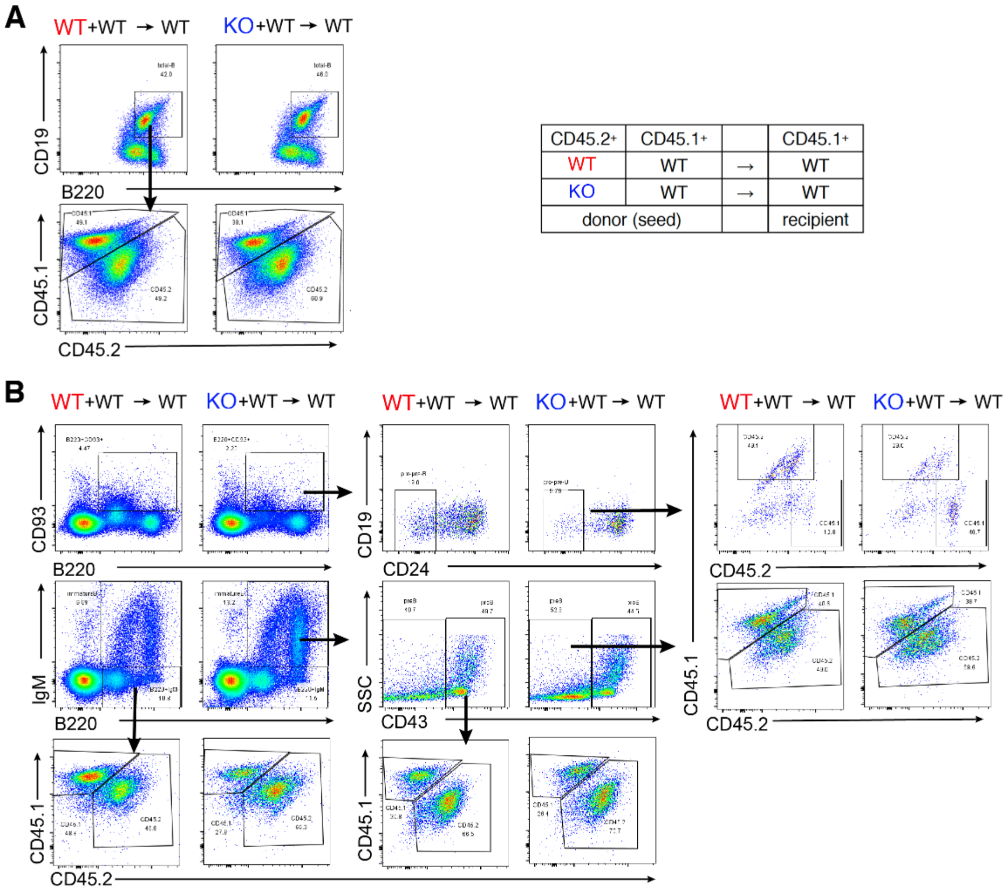

CD45.2+	CD45.1+		CD45.1+
WT	WT	→	WT
KO	WT	→	WT
donor (seed)			recipient

Additional file 3. Fig. S2. Representative flow analysis diagrams of *in vivo* bone marrow transplantation experiment (supplementary for Fig. 1J).
