# Supplementary material for: Lactoferrin is required for early B cell development in C57BL/6 mice
Source: J Hematol Oncol. 2021 Apr 7;14:58. doi: 10.1186/s13045-021-01074-6 (PMC8028198; doi:10.1186/s13045-021-01074-6)
Supplement: Supplementary file 4 — Additional file 4: Fig. S3. Lactoferrin deficiency alters genes expression profile and key pathways in pre-pro-B cells. [file 13045_2021_1074_MOESM4_ESM.pdf]

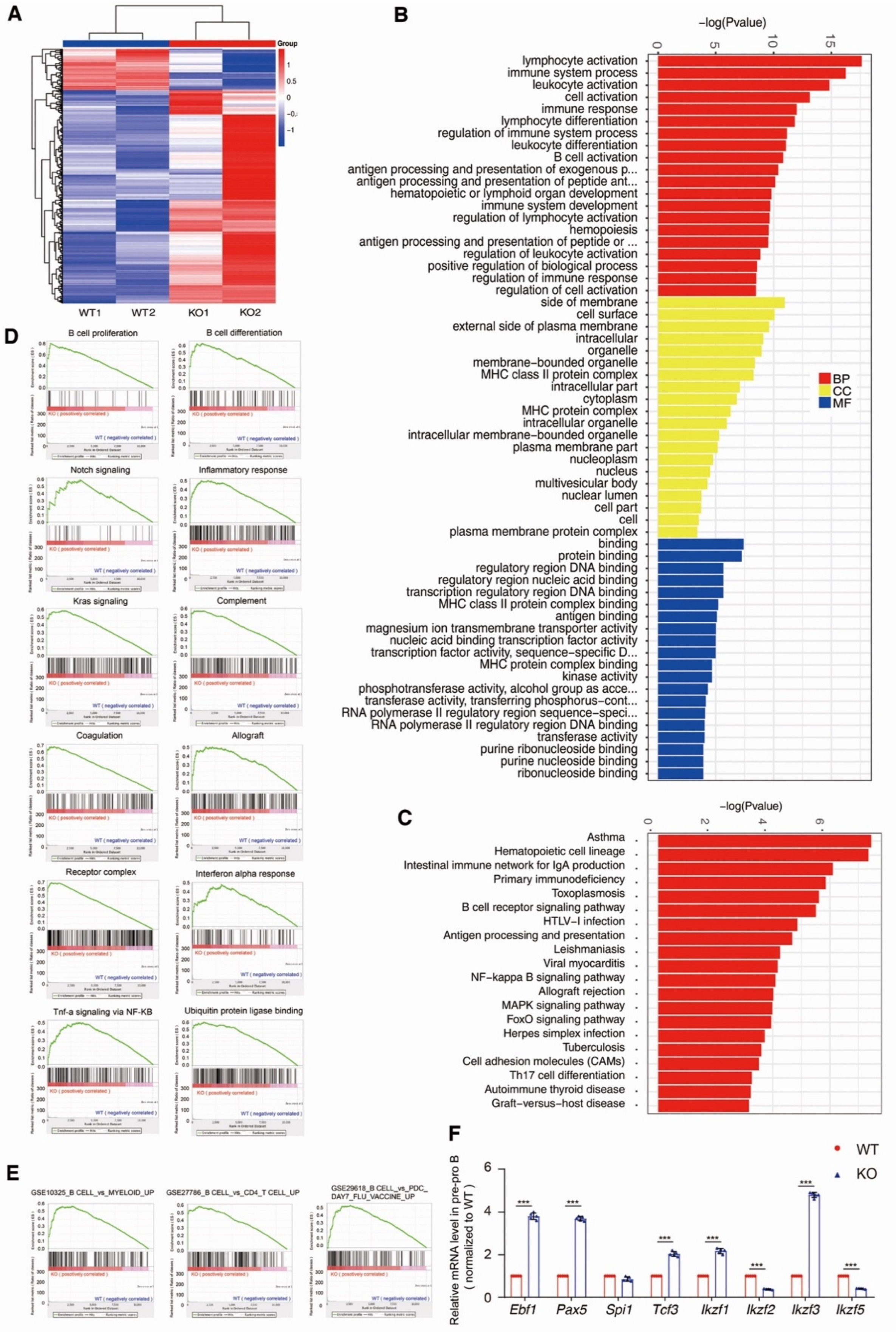

A
Group
1
0.5
0
-1
WT1
WT2
KO1
KO2
B
-log(Pvalue)
0
5
10
15
lymphocyte activation
immune system process
leukocyte activation
cell activation
immune response
lymphocyte differentiation
regulation of immune system process
leukocyte differentiation
B cell activation
antigen processing and presentation of exogenous p...
antigen processing and presentation of peptide ant...
hematopoietic or lymphoid organ development
immune system development
regulation of lymphocyte activation
hemopoiesis
antigen processing and presentation of peptide or ...
regulation of leukocyte activation
positive regulation of biological process
regulation of immune response
regulation of cell activation
side of membrane
cell surface
external side of plasma membrane
intracellular
organelle
membrane-bounded organelle
MHC class II protein complex
intracellular part
cytoplasm
MHC protein complex
intracellular organelle
intracellular membrane-bounded organelle
plasma membrane part
nucleoplasm
nucleus
multivesicular body
nuclear lumen
cell part
cell
plasma membrane protein complex
binding
protein binding
regulatory region DNA binding
regulatory region nucleic acid binding
transcription regulatory region DNA binding
MHC class II protein complex binding
antigen binding
magnesium ion transmembrane transporter activity
nucleic acid binding transcription factor activity
transcription factor activity, sequence-specific D...
MHC protein complex binding
kinase activity
phosphotransferase activity, alcohol group as acce...
transferase activity, transferring phosphorus-cont...
RNA polymerase II regulatory region sequence-speci...
RNA polymerase II regulatory region DNA binding
transferase activity
purine ribonucleoside binding
purine nucleoside binding
ribonucleoside binding
BP
CC
MF
C
-log(Pvalue)
0
2
4
6
Asthma
Hematopoietic cell lineage
Intestinal immune network for IgA production
Primary immunodeficiency
Toxoplasmosis
B cell receptor signaling pathway
HTLV-I infection
Antigen processing and presentation
Leishmaniasis
Viral myocarditis
NF-kappa B signaling pathway
Allograft rejection
MAPK signaling pathway
FoxO signaling pathway
Herpes simplex infection
Tuberculosis
Cell adhesion molecules (CAMs)
Th17 cell differentiation
Autoimmune thyroid disease
Graft-versus-host disease
D
B cell proliferation
B cell differentiation
Notch signaling
Inflammatory response
Kras signaling
Complement
Coagulation
Allograft
Receptor complex
Interferon alpha response
Tnf-a signaling via NF-KB
Ubiquitin protein ligase binding
KO (positively correlated)
WT (negatively correlated)
E
GSE10325_B CELL_vs_MYELOID_UP
GSE27786_B CELL_vs_CD4_T CELL_UP
GSE29618_B CELL_vs_PDC_DAY7_FLU_VACCINE_UP
F
WT
KO
Relative mRNA level in pre-pro B (normalized to WT)
0
2
4
6
***
Ebf1
Pax5
Spi1
Tcf3
Ikzf1
Ikzf2
Ikzf3
Ikzf5

Additional file 4 Fig. S3. Lactoferrin deficiency alters genes expression profile and key pathways in pre-pro-B cells. **(A)** Hierarchical clustering of transcripts that were upregulated or downregulated in pre-pro-B cells of $Lf^{-/-}$ mice comparing to that of WT controls, with two repeats. **(B)** GO enrichment analysis was used to analyze the biological functions of the differently expressed genes between WT and $Lf^{-/-}$ pre-pro-B cells. The category includes biological process (BP), cellular component (CC) and molecular function (MF). The y-axis is enrichment score, and the x-axis is enriched GOs. **(C)** KEGG pathways related to the differently expressed genes between WT and $Lf^{-/-}$ pre-pro-B cells. The top 20 positively enriched pathways were shown in histogram. **(D)** Gene sets difference between WT and $Lf^{-/-}$ pre-pro-B cells revealed by GSEA. 12 representative enriched biological pathway gene sets were listed here. **(E)** Expression spectrum data of mice were analyzed with GESA of 4872 immunologic signatures sets, and the listed is the top gene sets enriched in $Lf^{-/-}$ pre-pro-B cells comparing to WT control. **(F)** The expression levels of a panel of B cell development-related transcription factors in WT and $Lf^{-/-}$ pre-pro-B cells were determined by RT-qPCR.
