# Supplementary material for: Lactoferrin is required for early B cell development in C57BL/6 mice
Source: J Hematol Oncol. 2021 Apr 7;14:58. doi: 10.1186/s13045-021-01074-6 (PMC8028198; doi:10.1186/s13045-021-01074-6)
Supplement: Supplementary file 5 — Additional file 5: Fig. S4. Lactoferrin deficiency alters genes expression profile and key pathways in pro-B cells. [file 13045_2021_1074_MOESM5_ESM.pdf]

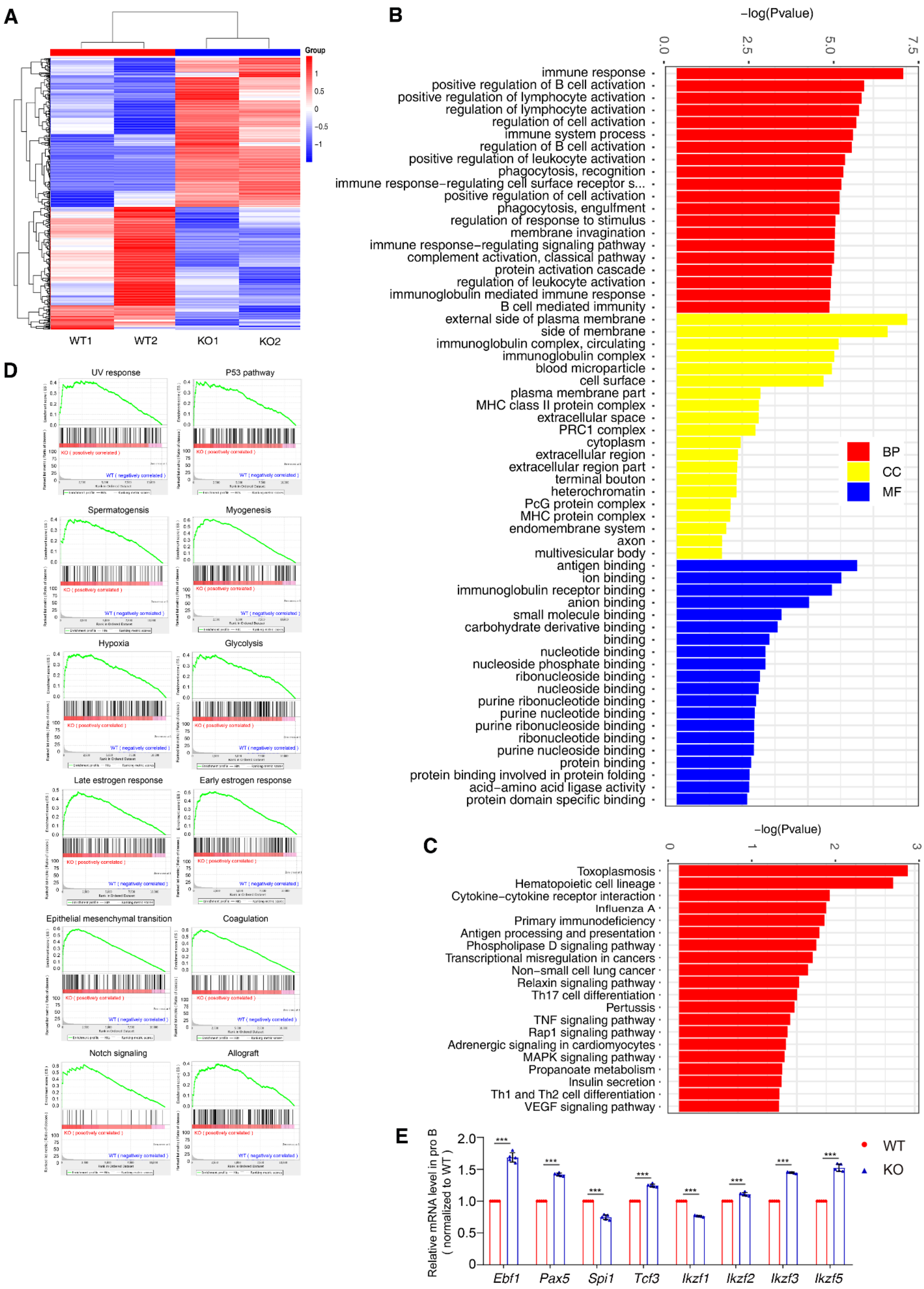

Additional file 5. Fig. S4. Lactoferrin deficiency alters genes expression profile and key pathways in pro-B cells. (A) Hierarchical clustering of transcripts that were upregulated or

downregulated in pro-B cells of *Lf*$^{-/-}$ mice comparing to that of WT controls, with two repeats. **(B)** GO enrichment analysis was used to analyze the biological functions of the differently expressed genes between WT and *Lf*$^{-/-}$ pro-B cells. The category includes biological process (BP), cellular component (CC) and molecular function (MF). The y-axis is enrichment score, and the x-axis is enriched GOs. **(C)** KEGG pathways related to the differently expressed genes between WT and *Lf*$^{-/-}$ pro-B cells. The top 20 positively enriched pathways were shown in histogram. **(D)** Gene sets difference between WT and *Lf*$^{-/-}$ pro-B cells revealed by GSEA. 12 representative enriched biological pathway gene sets were listed here. A gene set with nominal $p \leq 0.05$ was considered to be significantly enriched. **(E)** The expression levels of a panel of B cell development-related transcription factors in WT and *Lf*$^{-/-}$ pro-B cells were determined by RT-qPCR.
