# Supplementary material for: Lactoferrin is required for early B cell development in C57BL/6 mice
Source: J Hematol Oncol. 2021 Apr 7;14:58. doi: 10.1186/s13045-021-01074-6 (PMC8028198; doi:10.1186/s13045-021-01074-6)
Supplement: Supplementary file 6 — Additional file 6: Fig. S5. Effect of lactoferrin deficiency on Cxcl2 expression. [file 13045_2021_1074_MOESM6_ESM.pdf]

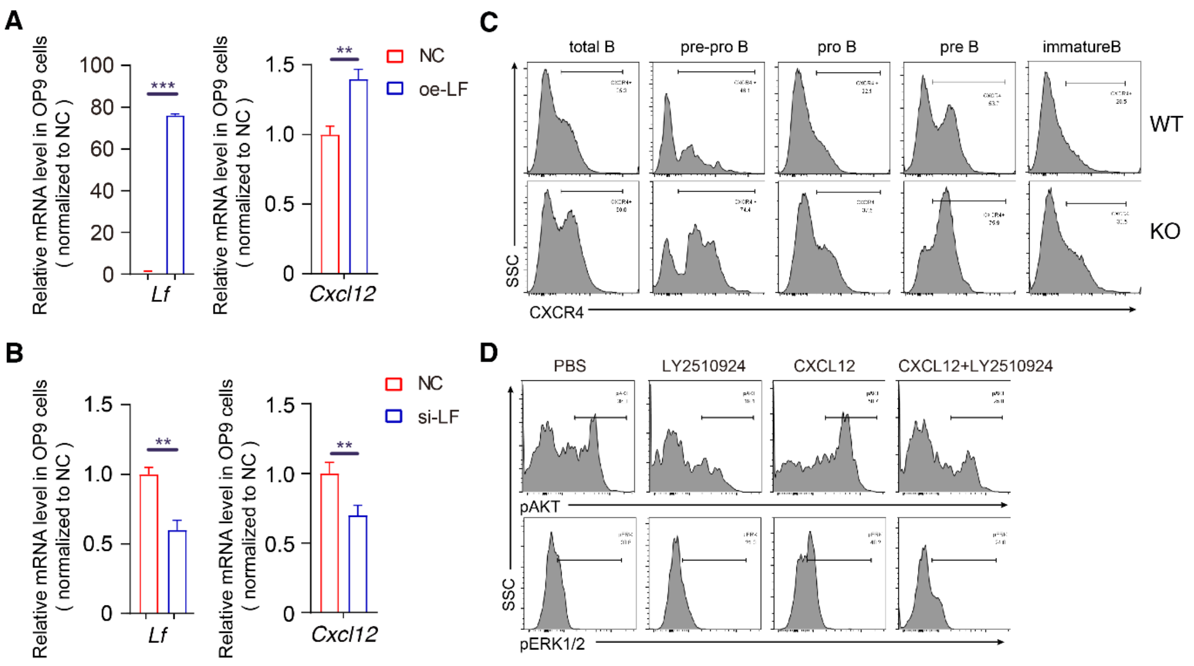

Additional file 6. Fig. S5. Effect of lactoferrin deficiency on Cxcl2 expression. (A) Lactoferrin re-expression in OP9 cells, and the expression levels of lactoferrin and CXCL12 were determined by RT-qPCR. **(B)** Si-RNA induced lactoferrin inhibition in OP9 cells, and the expression levels of lactoferrin and CXCL12 were determined by RT-qPCR. (Supplementary for Fig. 2). **(C)** Different stages of B cells were isolated from the WT and $Lf^{-/-}$ mouse bone marrow cells, and the surface expression of CXCR4 in each stage of B cells was determined by flow cytometric analysis. Representative flow analysis diagrams of distribution of CXCR4 on the surface of B cells (Supplementary for Fig. 2F). **(D)** *In vitro* differentiation experiment: bone marrow cells from $Lf^{-/-}$ mice were added in 12-well plates at 5×10^4 cells per well, with (1) PBS, or (2) LY2510924 (10 ng/ml), or (3) CXCL12 recombinant protein (10 ng/ml), or (4) LY2510924 (10 ng/ml) and CXCL12 recombinant protein (10 ng/ml), for 9 days. Representative flow analysis diagrams of distribution of p-ERK1/2 and p-AKT in B cells (Supplementary for Fig. 2H).
