# Supplementary material for: Lactoferrin is required for early B cell development in C57BL/6 mice
Source: J Hematol Oncol. 2021 Apr 7;14:58. doi: 10.1186/s13045-021-01074-6 (PMC8028198; doi:10.1186/s13045-021-01074-6)
Supplement: Supplementary file 7 — Additional file 7: Fig. S6. Lactoferrin deficiency affects the proportion of splenic B cells subclasses and antibody production in B cells. [file 13045_2021_1074_MOESM7_ESM.pdf]

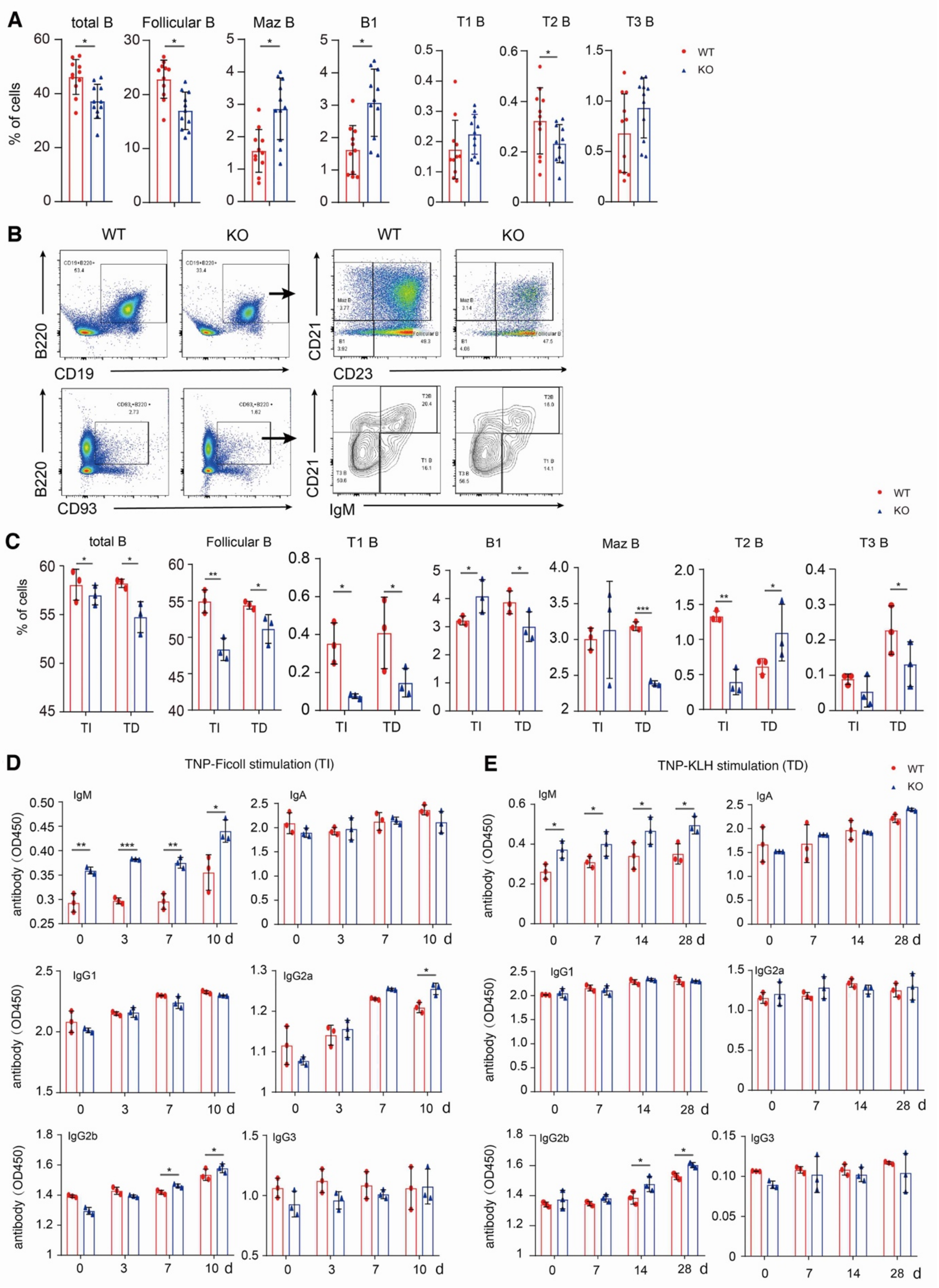

Additional file 7. Fig. S6. Lactoferrin deficiency affects the proportion of splenic B cells subclasses and antibody production in B cells. **(A)** Splenic B cells from WT or $Lf^{-/-}$ mice were sorted. Frequencies of total B cells ($CD19^{+}B220^{+}$), follicular B cells ($CD19^{+}B220^{+}CD23^{+}CD21^{+}$),

marginal B cells ($CD19^+B220^+CD23^- CD21^+$), B1 cells ($CD19^+B220^+CD23^- CD21^-$), T1 B cells ($B220^+CD93^+IgM^+CD23^-$), T2 B cells ($B220^+CD93^+IgM^+CD23^+$), and T3 B cells ($B220^+CD93^+IgM^-CD23^-$) cells were identified by flow cytometry. Each group has 11 mice. **(B)** Representative flow analysis diagrams of splenic B cells from WT and $Lf^{-/-}$ mice. **(C)** Frequencies of total B, follicular B, B1 cells, marginal B cells, T1 B cells, T2 B cells and T3 B cells from WT and $Lf^{-/-}$ mice (n=3) upon stimulation by TNP-Ficoll (TI) or TNP-KLH (TD). **(D)** ELISA was performed for TNP-specific immunoglobulins of serum in TNP-Ficoll immunized mice (n=3). **(E)** ELISA was performed for TNP-specific immunoglobulins of serum in TNP-KLH immunized mice (n=3).
