# Supplementary material for: Lactoferrin is required for early B cell development in C57BL/6 mice
Source: J Hematol Oncol. 2021 Apr 7;14:58. doi: 10.1186/s13045-021-01074-6 (PMC8028198; doi:10.1186/s13045-021-01074-6)
Supplement: Supplementary file 8 — Additional file 8: Fig. S7. Lactoferrin deficiency promotes the progression of SLE in mice. [file 13045_2021_1074_MOESM8_ESM.pdf]

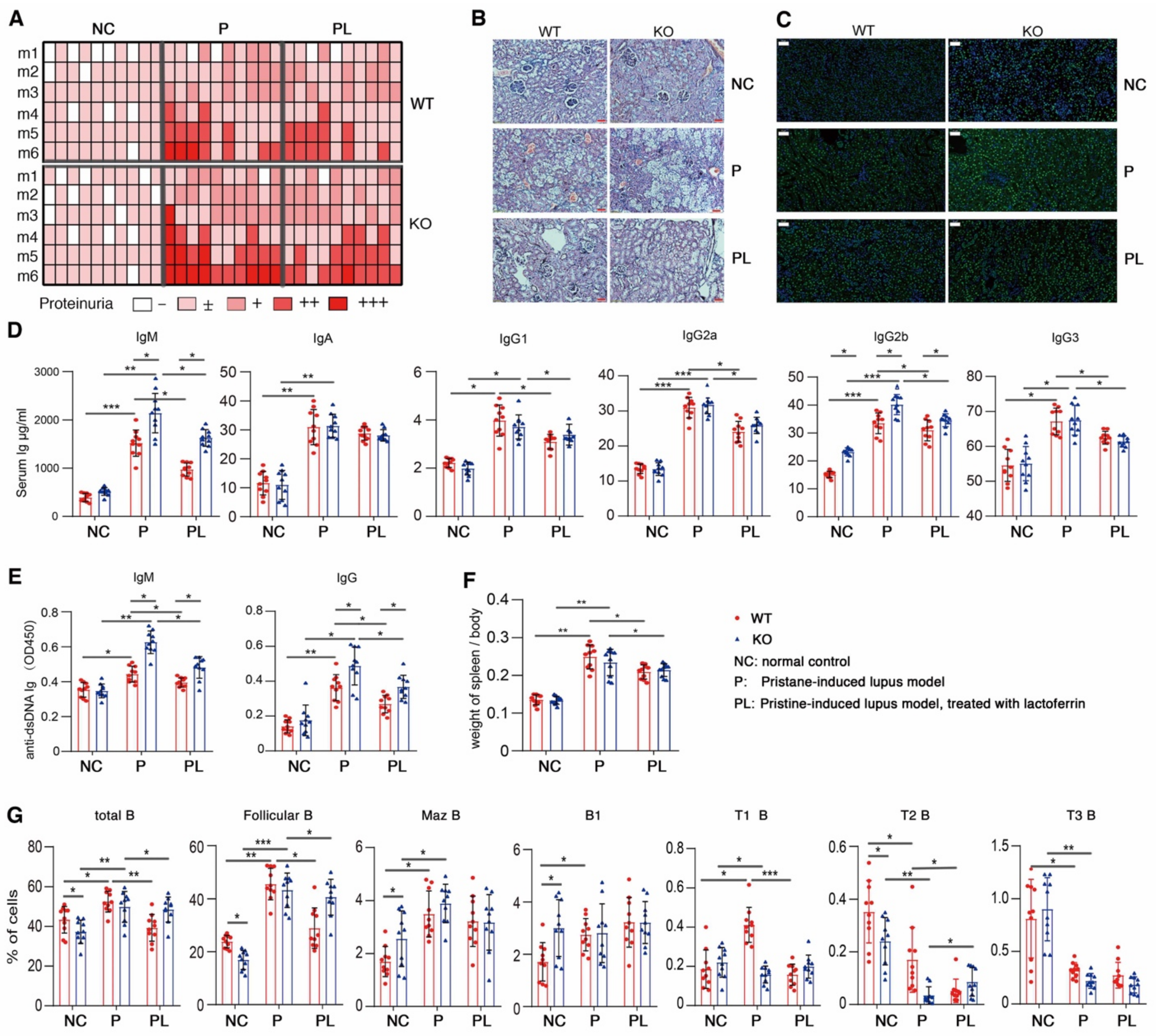

Additional file 8. Fig. S7. Lactoferrin deficiency promotes the progression of SLE in mice.

(A) The positive rate of proteinuria and the amount of proteinuria from month 0 (m0) to months 6 (m6) during the process in pristane-induced SLE mouse model. The mice had been divided into three groups: (1) normal control (NC), injected with saline solution, (2) pristane-injection induced SLE model, (3) pristane-injection, with oral lactoferrin treatment. Each group has 10 mice. **(B)** Representative images of renal hematoxylin/eosin staining. Scale bar: 50 μm. **(C)** Representative images of renal immune-fluorescence of IgG antibodies. Scale bar: 50 μm. **(D)** ELISA was performed for immunoglobulins of serum of mice. **(E)** ELISA was performed for anti-dsDNA IgG and anti-dsDNA IgM of serum of mice. **(F)** The weight ratio of spleen vs body in mice. **(G)** Frequencies of total B cells and the subclass cells were identified by flow cytometry. All data were in a one-way analysis of variance followed by Bonferroni correction.
