# Supplementary material for: Lactoferrin is required for early B cell development in C57BL/6 mice
Source: J Hematol Oncol. 2021 Apr 7;14:58. doi: 10.1186/s13045-021-01074-6 (PMC8028198; doi:10.1186/s13045-021-01074-6)
Supplement: Supplementary file 9 — Additional file 9: Table S1. Information on the antibodies used in FACS or mice experiments. [file 13045_2021_1074_MOESM9_ESM.pdf]

Additional file 9. Table S1. Information on the antibodies used in FACS or mice experiments

Antibody	Fluorescent	Catalog #	Dilution	Company
anti-mouse CD93	PE/Cy7	136505	1:100	Biolegend
anti-mouse CD31	PE	102407	1:100	Biolegend
anti-mouse Ter-119	APC	116211	1:100	Biolegend
anti-mouse CD106	Percp-Cy5.5	105715	1:100	Biolegend
anti-mouse B220	PE/Cy7	103222	1:100	Biolegend
anti-mouse CD45	FITC	103107	1:100	Biolegend
anti-mouse AKT (pS473)	PE	560378	1:100	BD
anti-mouse ERK1/2	PE/Cy7	560116	1:100	BD
anti-mouse CD45.1	FITC	110705	1:100	Biolegend
anti-mouse CD45.2	BV510	109837	1:100	Biolegend
anti-mouse IgM	PE	406507	1:100	Biolegend
anti-mouse CD19	APC/Cy7	115530	1:100	Biolegend
anti-mouse B220	Percp-Cy5.5	103236	1:100	Biolegend
anti-mouse CD43	APC	143207	1:100	Biolegend
anti-mouse CD24	FITC	101805	1:100	Biolegend
anti-mouse CD184 (CXCR4)	PE/Dazzle	146513	1:100	Biolegend
anti-mouse CD4	APC	100411	1:100	Biolegend
anti-mouse F4/80	APC	123115	1:100	Biolegend
anti-mouse MHCII	Percp-Cy5.5	107625	1:100	Biolegend
anti-mouse CD45	PE/Dazzle 594	103145	1:100	Biolegend
anti-mouse CD21	BV421	123421	1:100	Biolegend
anti-mouse Lin	Alexa Fluov 700	79923	1:100	Biolegend
anti-mouse Lin	FITC	78022	1:100	Biolegend
anti-mouse CD11c	BV421	117329	1:100	Biolegend
anti-mouse 117	PE	105807	1:100	Biolegend
anti-mouse CD93	PE	136503	1:100	Biolegend
anti-mouse CD11b	PE	101207	1:100	Biolegend
anti-mouse CD3e	FITC	100305	1:100	Biolegend
anti-mouse CD8a	PE/Cy7	100707	1:100	Biolegend
anti-mouse NK1.1	PE	108713	1:100	Biolegend
anti-mouse CD23	APC	101619	1:100	Biolegend
anti-mouse Sca-1	Percp-Cy5.5	108105	1:100	Biolegend
