# Supplementary material for: Lactoferrin is required for early B cell development in C57BL/6 mice
Source: J Hematol Oncol. 2021 Apr 7;14:58. doi: 10.1186/s13045-021-01074-6 (PMC8028198; doi:10.1186/s13045-021-01074-6)
Supplement: Supplementary file 10 — Additional file 10: Table S2. Antibodies information for ELISA. [file 13045_2021_1074_MOESM10_ESM.pdf]

Additional file 10. Table S2. Antibodies information for ELISA

Reagents	Catalog #	Company
Mouse CXCL12 (SDF-1) pre-coated ELISA Kit	444207	Biolegend
Ancillary Reagent Kit	DY008	R&D
Purified Rat Anti-mouse IgA	556960	BD
Purified Rat Anti-mouse IgG2b	553392	BD
Purified Rat Anti-mouse IgM	553405	BD
Purified Rat Anti-mouse IgG2a	553446	BD
Purified Rat Anti-mouse IgG1	553445	BD
Purified Rat Anti-mouse IgG3	553404	BD
Mouse IgG3 Antibody	A90-111P	Biolegend
Mouse IgG2b Antibody	A90-109P	Biolegend
Mouse IgA Antibody	A90-103P	Biolegend
Mouse IgG1 Antibody	A90-105P	Biolegend
Mouse IgM Antibody	A90-101P	Biolegend
Mouse IgG2a Antibody	A90-107P	Biolegend
ELISA Kits for Proteins, Hormones, Steroids and IgG's Mouse Autoimmune Kits (mouse anti-dsDNA IgM)	190726K2	Alpha diagnostic international
ELISA Kits for Proteins, Hormones, Steroids and IgG's Mouse Autoimmune Kits (mouse anti-dsDNA IgG)	190726K3	Alpha diagnostic international
Mouse IL-7 DuoSet ELISA	DY407	R&D
