# Supplementary material for: Lactoferrin is required for early B cell development in C57BL/6 mice
Source: J Hematol Oncol. 2021 Apr 7;14:58. doi: 10.1186/s13045-021-01074-6 (PMC8028198; doi:10.1186/s13045-021-01074-6)
Supplement: Supplementary file 11 — Additional file 11: Table S3. Primer sequences used for qPCR. [file 13045_2021_1074_MOESM11_ESM.pdf]

Additional file 11. Table S3. Primer sequences used for qPCR

**Genes**	**Forward Sequence**	**Reverse Sequence**
Lf	TGA GGC CCT TGG ACT CTG T	ACC CAC TTT TCT CAT CTC GTT C
Il4	TGG TGG GAG ACT TAC CTG ATG	CCC GGA TAA CTT CAC AAC TTC TT
Il7	GCG GAC GAT CAC TCC TTC TG	AGC CCC ACA TAT TTG AAA TTC CA
Il12	AGA GAA TGC TCA TTG GCA CTT C	AAC TGG GAT AAT GTG AAC AGC C
Spi1	AGG AGT CTT CTA CGA CCT GGA	GAA GGC TTC ATA GGG AGC GAT
Il15	AAT CAG ATA CCG CAA TGA CCA C	CAG AAG TTG TTT GGG ATG GTG T
Cxcl12	TGC ATC AGT GAC GGT AAA CCA	TTC TTC AGC CGT GCA ACA ATC
Csf1	GAC CCT GAA TCT CCC GGA AG	GGT ACA ACG GTA GGT CCC AG
Ebf1	GCA GCC ACC ATC TAG CCT G	CAG CAG TGA GTC TGC CTT GAT
Tcf3	GGG TGC CAG CGA GAT CAA G	ATG AGC AGT TTG GTC TGC GG
Ikzf1	AGA CAA GTG CCT GTC AGA CAT	CCA GGT AGT TGA TGG CAT TGT TG
Ikzf2	GAG CCG TGA GGA TGA GAT CAG	CTC CCT CGC CTT GAA GGT C
Ikzf3	CTG AAT GAC TAC AGC TTG CCC	GCT CCG GCT TCA TAA TGT TCT
Ikzf5	CCA GAG CCT TTG GAC TTT GTA A	AAC TGA TCC CGA AAT CAT GTT CA
Pax5	CCA TCA GGA CAG GAC ATG GAG	GGC AAG TTC CAC TAT CCT TTG G
Gapdh	AAT GGA TTT GGA CGC ATT GGT	TTT GCA CTG GTA CGT GTT GAT
